# Leptospirosis Infection Misdiagnosed as COVID-19: A Rare Case Report

**DOI:** 10.7759/cureus.29106

**Published:** 2022-09-13

**Authors:** Hüseyin Elçi, Özhan Orhan

**Affiliations:** 1 Pediatrics, Kızıltepe State Hospital, Mardin, TUR

**Keywords:** differential diagnosis, case report, leptospirosis, pediatric infectious disease, covid-19

## Abstract

Leptospirosis is an acute, febrile, systemic, and zoonotic infectious disease characterized by widespread vasculitis caused by *Leptospira interrogans* from the leptospira family. It can be in the form of asymptomatic infection; it can also progress with severe symptomatic forms characterized by multiorgan involvement such as aseptic meningitis as well as liver and kidney failure. Leptospirosis is transmitted to humans through water, soil, and food contaminated with the urine of infected mice or other mammals.

COVID-19 is a newly detected coronavirus that causes pneumonia. The disease has led to a pandemic all over the world. In this case report, we aimed to draw attention to leptospirosis infection in the presence of a case who was followed up with the differential diagnosis of COVID-19 and diagnosed with leptospirosis during the COVID-19 pandemic. Leptospirosis is one of the diagnoses that should be kept in mind in especially developing countries in patients presenting with findings that may be confused with COVID-19 during the pandemic period.

## Introduction

Leptospirosis is an acute, febrile, systemic, and zoonotic infectious disease characterized by widespread vasculitis caused by *Leptospira interrogans* from the leptospira family [1]. It can be in the form of asymptomatic infection, which can also progress with severe symptomatic forms characterized by multiorgan involvement such as aseptic meningitis as well as liver and kidney failure. Symptomatic infection is seen as anicteric in 90% and severe icteric (Weil syndrome) in 5%-10% [2]. Leptospirosis is transmitted to humans through water, soil, and food contaminated with the urine of infected mice or other mammals [2,3]. Farmers, veterinarians, slaughterhouse workers, miners, sewers, swimmers, and campers constitute risk groups for the disease [4,5]. Leptospirosis, which is still an important public health problem in developing countries, is rarely seen in developed countries.

In the process that started with the reporting of pneumonia cases of unknown cause in the city of Wuhan, located in the Hubei province of China, in December 2019, a new coronavirus was detected as a pneumonia agent, and the disease caused by the virus was named “coronavirus disease 2019” (COVID-19) [6]. The disease has led to a pandemic all over the world.

In this case report, we aimed to draw attention to leptospirosis infection in the presence of a case who was followed up with the differential diagnosis of COVID-19 and diagnosed with leptospirosis during the COVID-19 pandemic.

This article was previously presented as a poster at the 2021 Turkish Pediatric Congress on October 19, 2021.

## Case presentation

A three-year, four-month-old male patient, who had no known disease before, presented with complaints of cough lasting for two days, fever reaching 40°C, weakness, and abdominal pain. Since our patient had a history of domestic contact, he was hospitalized in the COVID-19 reserved part of the hospital with a prediagnosis of COVID-19.

At the time of admission, the patient's temperature was 38.5°C, heart rate was 130/min, respiratory rate was 32/min, lung sounds were normal, and abdominal examination was normal. There was no organomegaly, and other system examinations were normal. In laboratory findings, leukocytes (WBC) were 9060/mm^3^, hemoglobin (Hgb) was 13.2 g/dL, hematocrit (Hct) was 41%, platelet count was 143 000/mm^3^, urea was 14 mg/dL, creatinine was 0.87 mg/dL, alanine aminotransferase (ALT) was 11 IU /L, aspartate aminotransferase (AST) was 26 IU/L, and C-reactive protein (CRP) was 148 mg/L.

Pneumonic infiltration compatible with COVID-19 was not detected in the lung x-ray of the patient, and thoracic CT was not performed due to the patient's findings and age. No hematological disease was considered in the evaluation of the peripheral smear. The patient was started on hydration and cefotaxime (150 mg/kg/day). The patient’s personal and family history was uncharacteristic. It was learned that the family of the patient was engaged in farming in the village, and the day after admission, doxycycline (2 mg/kg/day for seven days) was added to the treatment considering leptospirosis. The leptospirosis PCR test, which was sent to the health directorate, was positive. The COVID PCR tests, which were sent two times, were negative. Abdominal ultrasonography (USG) was evaluated as normal. There was no growth in the blood culture taken during the febrile period. In the follow-up of the patient, icterus did not develop. On the fourth day, the complaints passed, and CRP regressed. The patient was discharged after the seven-day treatment was completed.

## Discussion

Leptospirosis has a wide distribution all over the world. The disease is more common in summer and early autumn. An average of 50-100 cases have been reported annually in the United States for the last 20 years [1]. In a study involving 700 workers in paddy fields in the Eastern Mediterranean parts of our country, antibody positivity against leptospira antigen was found at a rate of 13% [7]. Serological studies in humans reveal that a large number of subclinical infections occur. Serological screening of veterinarians and slaughterhouse workers has demonstrated positive titers in 5%-16% of people tested [8-10]. Leptospires remain resistant in the kidney tubules for a long time and are excreted in their urine without causing any disease in many animals, and even immunized dogs expel leptospires in their urine for a long time. At the same time, the constant reinfection of wild animals in the pet community are two main problems that complicate the effective control of leptospirosis [1]. Indirect contact, especially through soil, water, and nutrients contaminated with the urine of infected mice, is the most common mode of transmission than direct contact with an infected animal. Our patient is three years and four months old. Infection is seen in farmers who are often engaged in irrigation in the south east of Turkey, and our patient's family was also engaged in irrigation in a village.

The fact that our case was in the period of COVID-19 pandemic, had a history of domestic contact for COVID-19, and fever, cough, weakness, abdominal pain, and high CRP caused COVID-19 to be considered in the differential diagnosis. However, this diagnosis was dismissed due to negative lung x-ray and COVID-19 PCR tests, which were within normal limits.

Leptospires enter the human body through the oral, nasopharyngeal, vaginal mucosa, and conjunctiva or through the skin with impaired integrity [1,2,11]. The disease has a two-period course after an incubation period of seven to 12 days (maximum of two to 20 days). The onset period (septicemic or leptospiremic period) begins abruptly and usually lasts four to seven days. In this period, which is characterized by nonspecific signs and symptoms, high fever; chills; severe muscle ache, especially in the calf, back, abdomen, and neck regions; headaches; weakness; nausea, and vomiting are observed. The child is typically lethargic [1,11]. The most characteristic finding in this period is conjunctival hemorrhage associated with photophobia. There are erythematous, maculopapular, sometimes, petechial, and purpuric skin eruptions, especially on the trunk, in 10%. During this period, leptospires can be isolated from blood, cerebrospinal fluid (CSF), and many body tissues. Our diagnosis was based on the PCR test performed on the blood sample as well as the clinical findings.

The second period (immune or leptospiruric period) lasts for four to 30 days. In anicteric forms, an afebrile recovery period of one to two days is observed in between. Fever and initial symptoms continue. This period is characterized by the presence of IgM-type antibodies in the circulation. Leptospires disappear from the blood and CSF during this period; but they can be seen resistant in the kidney, urine, and eye fluid [1]. It is thought that cytokines such as tumor necrosis factor and interleukins 1 and 6 released during rapid phagocytosis of *L. interrogans* mediate the emergence of symptoms, especially second-stage symptoms [3]. The primary lesion seen in the liver, kidney, muscle, and meninges in leptospirosis is vasculitis, possibly due to the damage to the endothelial layer of small blood vessels, induced by leptospiral toxins, resulting in ischemic injury [1,2]. For this reason, in all systems where vasculitis can be seen, failure of systems such as liver, kidney, and heart and clinical findings resulting in death may occur. Renal insufficiency is the leading cause of death. The microorganism can be produced in the blood and CSF in the first period and in the urine in the second period using semisolid media such as Fletcher's medium [1].

While jaundice seen in severe leptospirosis cases is due to primary hepatocellular dysfunction, hemolysis that occurs rarely in the course of the disease is another cause of jaundice [11]. Severe anemia is considered to be characteristic of leptospirosis with jaundice. Leptospirosis cases with icterus are considered Weil's syndrome. In our patient, transaminases were not elevated and jaundice did not develop. Therefore, our patient was not having Weil's syndrome but was interpreted as a case of anicteric leptospirosis form.

In the differential diagnosis of leptospirosis, meningitis, COVID-19, hepatitis, nephritis, fever of unknown origin, influenza, Kawasaki syndrome, toxic shock syndrome, and Legionella disease should be considered [1-3]. In our patient, these entities were ruled out with clinical and laboratory findings.

In the treatment of leptospirosis, it is recommended to start penicillins and tetracyclines as soon as the disease is suspected. While the presence of azotemia and jaundice requires careful liquid electrolyte treatment, peritoneal dialysis may be required in patients with azotemia, and exchange transfusion may be required in patients with high hyperbilirubinemia [3]. We applied cefotaxime, doxycycline, and fluid therapy to our patient.

In the prevention of leptospirosis, disinfection of contaminated areas and waters, prevention of swimming in contaminated waters, and control of mice and other rodents are required [12]. Vaccination of farm animals and pets has limited usefulness as humans can become infected even from vaccinated animals. Today, polyvalent human vaccines are administered in the risk regions of Europe and Asia [3]. It has been reported that prophylactic doxycycline treatment (200 mg/week) applied to those traveling to endemic areas is also beneficial [13]. Due to the frequent occurrence of the disease in the south east of Turkey, a study was planned for its causes and prevalence, and the relevant institutions were informed.

## Conclusions

Leptospirosis should be among the diseases considered in patients with acute, febrile, and multiple organ failure in the south east of Turkey. There is a large number of wild and domestic animals due to the widespread farming and geographical structure, but no vaccination programs are active for leptospirosis, and water and nutrient hygiene are insufficient. For this reason, leptospirosis is one of the diagnoses that should be kept in mind in this region in patients presenting with findings that may be confused with COVID-19 during the pandemic period.

Due to the frequent occurrence of the disease in the south east of Turkey, it is planned to conduct a study on its causes and prevalence. Relevant organizations have been informed about the necessary studies for its prevention.
